# Imaging features of the initial chest thin-section CT scans from 110 patients after admission with suspected or confirmed diagnosis of COVID-19

**DOI:** 10.1186/s12880-020-00464-5

**Published:** 2020-06-15

**Authors:** Cheng-Juan Long, Ping Fang, Tie-Jun Song, Jing-Chao Zhang, Qing Yang

**Affiliations:** Department of Medical Imaging, Anqing Hospital Affiliated to Anhui Medical University, Renmin road, Anqing, 246000 Anhui China

**Keywords:** Coronavirus, COVID-19, Pneumonia, Thin-section CT

## Abstract

**Background:**

In December 2019, an outbreak of a novel coronavirus pneumonia, now called COVID-19, occurred in Wuhan, Hubei Province, China. COVID-19, which is caused by the severe acute respiratory syndrome coronavirus 2 (SARS-CoV-2), has spread quickly across China and the rest of the world. This study aims to evaluate initial chest thin-section CT findings of COVID-19 patients after their admission at our hospital.

**Methods:**

Retrospective study in a tertiary referral hospital in Anhui, China. From January 22, 2020 to February 16, 2020, 110 suspected or confirmed COVID-19 patients were examined using chest thin-section CT. Patients in group 1 (*n* = 51) presented with symptoms of COVID-19 according to the diagnostic criteria. Group 2 (*n* = 29) patients were identified as a high degree of clinical suspicion. Patients in group 3 (*n* = 30) presented with mild symptoms and normal chest radiographs. The characteristics, positions, and distribution of intrapulmonary lesions were analyzed. Moreover, interstitial lesions, pleural thickening and effusion, lymph node enlargement, and other CT abnormalities were reviewed.

**Results:**

CT abnormalities were found only in groups 1 and 2. The segments involved were mainly distributed in the lower lobes (58.3%) and the peripheral zone (73.8%). The peripheral lesions, adjacent subpleural lesions, accounted for 51.8%. Commonly observed CT patterns were ground-glass opacification (GGO) (with or without consolidation), interlobular septal thickening, and intralobular interstitial thickening. Compared with group 1, patients in group 2 presented with smaller lesions, and all lesions were distributed in fewer lung segments. Localized pleural thickening was observed in 51.0% of group 1 patients and 48.2% of group 2 patients. The prevalence of lymph node enlargement in groups 1 and 2 combined was extremely low (1 of 80 patients), and no significant pleural effusion or pneumothorax was observed (0 of 80 patients).

**Conclusion:**

The common features of chest thin-section CT of COVID-19 are multiple areas of GGO, sometimes accompanied by consolidation. The lesions are mainly distributed in the lower lobes and peripheral zone, and a large proportion of peripheral lesions are accompanied by localized pleural thickening adjacent to the subpleural region.

## Background

**Fig. 1:**
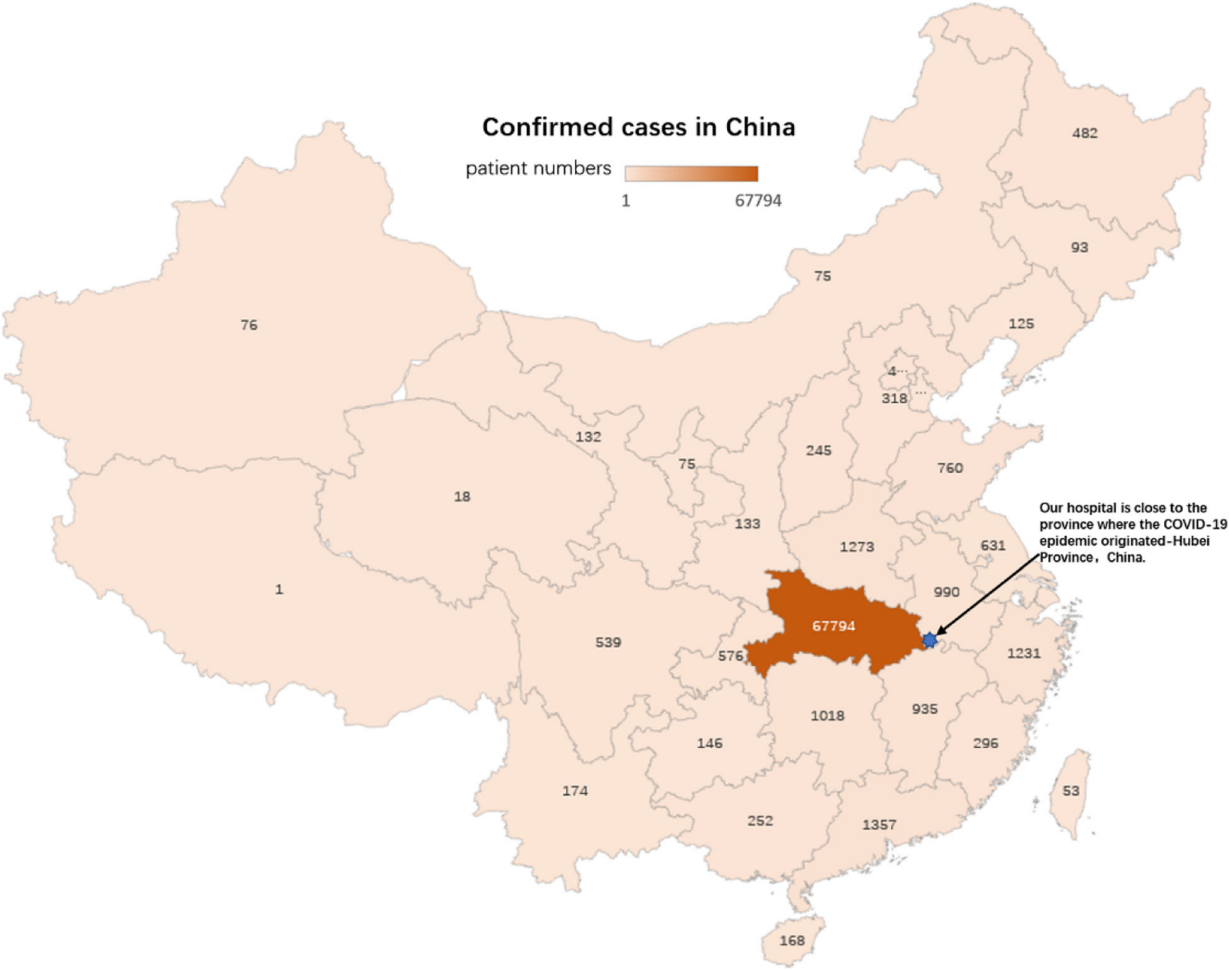
A statistical fusion map showing the cumulative number of confirmed COVID-19 patients in China by province (Map produced by: Y Qing; Source of data: WHO, March 15, 2020). The blue mark is the geographic location of our hospital

In December 2019, an outbreak of a novel coronavirus pneumonia, now called COVID-19, occurred in Wuhan, Hubei Province, China. COVID-19, which is caused by the severe acute respiratory syndrome coronavirus 2 (SARS-CoV-2), has spread quickly across China and the rest of the world, partly due to the massive migration of population associated with the Chinese Spring Festival. By March 15, 2020, there were a total of 81,048 confirmed cases in China, the vast majority of which (67,794) were in Hubei Province (Fig. 1), especially in Wuhan (49,999). A total of 72,469 confirmed cases have been reported worldwide (outside of China) [1]. Since January 22, 2020, when the first case of COVID-19was confirmed in our hospital’s infectious disease department in-patient ward, we have collaborated with the Chinese Center for Disease Control and Prevention (CCDC) and confirmed a total of 80 cases as of February 16, 2020, using viral nucleic acid test (NAT). The sharp increase in the number of confirmed cases may be partly due to the high infectivity of SARS-CoV-2 [2]. Another explanation might be that our hospital’s administrative zone borders Hubei Province (Fig. 1) with a distance of only 300 km from Wuhan City, the potential source of the disease, and there is a widespread migration of people between the two areas.

As described in the new provisional clinical diagnostic guidelines (fifth edition) of COVID-19 [3], developed by the National Health Commission of the People’s Republic of China in consideration of the World Health Organization (WHO) recommendations, the suspect criteria consist of the following two parts: (1) an epidemiological history of contact (i.e., a history of contact within 14 days) and (2) clinical manifestations, with the latter further consisting of three parts, as follows: (a) fever and/or respiratory symptoms; (b) a normal or decreased white blood cell count or a reduced lymphocyte count in the early stages of onset; and (c) CT abnormalities of COVID-19. As a suspect patient, the patient should either have an epidemiological history of contact and show two of the three clinical manifestations or have no clear epidemiological history of contact but show all three clinical manifestations. Based on the criteria for a patient suspected with COVID-19, the diagnostic criteria require that a given suspect patient should present at least one of the following two pathogenic evidence: (1) positive NAT for the SARS-CoV-2 in respiratory specimens or blood specimens through real-time fluorescent RT-PCR; and (2) high genetic sequence homology between the virus isolated from respiratory specimens or blood specimens and known coronaviruses. In this study, the diagnosed patients and suspect patients all conformed to the above diagnostic criteria and suspect criteria, respectively.Given that no specific test has yet been developed to diagnose this disease with adequate accuracy and reliability, the diagnosis must be based on clinical manifestations combined with the imaging features and epidemiological history of contact. According to the latest provisional guidelines developed by the National Health Commission of China considering WHO recommendations, chest X-ray examination results are part of the main diagnostic components [3]. A total of 110 patients were included in this study. The patients were divided into three groups. The symptoms and signs of group 1 (*n* = 51) patients were consistent with the diagnostic criteria developed by CCDC, and the chest X-ray images presented abnormal patterns. Group 2 (*n* = 29) patients were highly suspected of COVID-19 infection (consistent with the suspect criteria developed by CCDC), and their chest X-ray results were negative or temporarily unavailable. Patients in group 3 (*n* = 30) presented mild symptoms or were asymptomatic with only a contact history (a medical staff) or had a history of travel to the affected areas and thus were anxious. The objective of this study was to retrospectively analyze the imaging features of the first chest thin-section CT images obtained from patients diagnosed with COVID-19, who tested positive for the nucleic acid of SARS-CoV-2 at our hospital.

## Methods

### Patients and CT imaging equipment

This was a retrospective study; thus, informed consent was exempted by the institutional ethics committee. From January 22 to February 16, 2020, a total of 110 patients underwent high-resolution chest CT scans in our hospital. The patients were divided into three groups. Patients in group 1 (*n* = 51) were aged 16–75 years with an average age of 43.1 years, consisting of 40 males and 11 females. The symptoms and signs of group 1 patients were consistent with the diagnostic criteria developed by CCDC, and the chest X-ray images presented abnormal patterns. CT examination was performed at 0–4 days (2.8 days on average) after admission to the hospital. Patients in group 2 (*n* = 29) were aged 19–69 years with an average age of 41.3 years, and comprised 9 males and 20 females. The group 2 patients were highly suspected of COVID-19 infection (consistent with the suspect criteria developed by CCDC), and their chest X-ray results were negative or temporarily unavailable. CT examination was performed at 0–3 days (1.9 days on average) after admission. Patients in group 3 (*n* = 30, average age of 35.1 years) presented mild symptoms (mild fever or cough) or were asymptomatic with only a contact history (medical staff) or had a history of travel to the affected areas and thus were anxious. CT examination was performed at 0–9 days (3.4 days on average) after admission. All three groups of patients were subjected to NAT (at least twice) screening of the respiratory or blood specimens for SARS-CoV-2 using real-time fluorescent RT-PCR, and the results confirmed COVID-19 infection in groups 1 and 2, while confirming the absence of COVID-19 infection in group 3.

CT model and scanning parameters: A BrightSpeed Elite 16 CT scanner (General Electric, Milwaukee, WI, USA) was used; each patient underwent a complete chest CT scan under the following scanning parameters: beam collimation of 20 mm, detector configuration of 16 × 1.25, pitch of 1.375, rotation speed of 0.5 s, voltage of 120 kV, current of 40–130 mA (with the Smart mA technique for automatic modulation of the tube current), and noise index of 32. The patient was supine and subjected to scanning while inhaling. To better evaluate chest thin-section CT images, they were reconstructed using the Bone plus algorithm, with a reconstructed section thickness of 1.25 mm and a reconstructed section interval of 0.625 mm.

### Review of CT images

All images were interpreted by two radiologists with more than 10 years of diagnostic experience using an AW 4.4 S/W workstation (General Electric, Milwaukee, WI, USA), and conclusions were drawn for each image based on their discussion and consensus. CT features of intrapulmonary lesions were divided into three basic categories: ground-glass opacification (GGO) (defined as a hazy increased opacity of the lung parenchyma without obliteration of the underlying vascular structures), consolidation (defined as a hazy increased opacity of the lung parenchyma with obscuring of the underlying vascular structures), and a combination of GGO with consolidation. Lesions were divided into the following four size categories: small (< 1 cm in diameter), medium (1 cm < diameter < 3 cm), large (3 cm < diameter < 50% of the lung segment), and very large (diameter equal to 50–100% of the lung segment). Lesions were defined as peripheral lesions if they were distributed in the outer one-third of the lung field, otherwise, they were defined as central lesions. If lesions were spread and diffused, they were defined as central-peripheral lesions. Localized subpleural lesions were categorized as peripheral lesions and were defined as peripheral lesions that were located at a distance of less than 0.5 cm from the lesion edge to the subpleural areas. CT images were reviewed to identify whether there were air bronchogram, pleural effusion, and pneumothorax, as well as to calculate the number of enlarged lymph nodes. Moreover, attention was paid as to whether there were intralobular involvement, interlobular septal involvement, as well as pulmonary nodules, masses, cavities, and calcifications in the CT images.

## Results

All the patients included in this retrospective study underwent CT scanning for the first time after admission to our hospital. All cases in group 1 met the diagnostic criteria before CT. All cases in group 2 met the suspect criteria before CT and were finally diagnosed with COVID-19 through NAT on respiratory specimens. For data analysis, group 1 and group 2 were pooled to a new group M to represent a larger collection of patient samples diagnosed with COVID-19, consisting of 49 males and 31 females, ranging in age from 16 to 75 years, with an average age of 42.4 years. Group 3 consisted of 30 patients with an average age of 35.1 years, who were finally confirmed negative for COVID-19 after multiple rounds (at least twice) of NAT on respiratory specimens.

### Distribution of lesions

Data showed that all lobar segments of both lungs could be affected, but there was a significant difference in the distribution of lesions, that is, the main affected areas were the lower lobes (433 of 743 lesions in group M, accounting for 58.3%, Table 1). Among the 80 patients in group M, the number of patients with affected lower lobes (*n* = 75) was higher than those with other lobe involvement (50 with affected upper lobes, 40 with affected middle lobe or the lingula; the middle lobe or tongue was affected in 40 of 80 patients, Table 2). In addition, both groups 1 and 2 showed a higher number of lesions in the lower lobes (Table 1).

**Table 1:** Number of segments affected by abnormality

Location	No. of Segments		
	Group 1	Group 2	Total (Group M)
	(*n* = 562)	(*n* = 181)	(*n* = 743)
Upper lobe			
Apical			
Right	23	4	27
Left	NA	NA	
Posterior			
Right	31	10	41
Left	42	13	55
Anterior			
Right	23	9	32
Left	19	5	24
Total	138	41	179 (24.1%)
Middle lobe			
Medial	18	3	21
Lateral	22	7	29
Total	40	10	50 (6.7%)
Lingula			
Superior	27	5	32
Inferior	37	12	49
Total	64	17	81 (10.9%)
Lower lobe			
Superior			
Right	37	16	53
Left	35	11	46
Anterior basal			
Right	23	6	29
Left	26	9	35
Medial basal			
Right	29	9	38
Left	NA	NA	
Lateral basal			
Right	39	11	50
Left	38	16	54
Posterior basal			
Right	48	17	65
Left	45	18	63
Total	320	113	433 (58.3%)
Average per patient	11.0	6.2	9.3

*NA* not applicable; left lung is considered to contain an apicoposterior segment and an anteromedial basal segment rather than the separate segments as seen on the right

Note: Numbers in parentheses are percentages

**Table 2:** Number of patients with affected segments and number of lesions located in particular lung regions

Lesion Location	No. of Patients			No. of Lesions
	Group 1	Group 2	Total (Group M)	Total (Group M)
	(*n* = 51)	(*n* = 29)	(*n* = 80)	(*n* = 743)
Upper lobe	33	17	50	179 (24.1%)
Middle lobe or lingula	27	13	40	131 (17.6%)
Lower lobe	49	26	75	433 (58.3%)
Central	4	2	6	67 (9.0%)
Peripheral	42	25	67	548 (73.8%)
Peripheral-Near Subpleural	28	13	41	284 (38.2%)
Both central and peripheral	7	3	10	119 (16.0%)

Note: Number in parentheses are percentages

In group 1, 42 patients were affected in both lungs (*n* = 51), accounting for 82.3%, while the number of patients with involvement of only one lung was 9 (*n* = 51), accounting for 17.6%. Each patient had 1–15 affected lung segments with an average of 7.1 lung segments being affected (any segment with multiple lesions was counted only once). In group 2, the number of patients with both lungs affected was 23 (*n* = 29), accounting for 79.3%, while the number of patients with the involvement of only one lung was 6 (*n* = 29) accounting for 20.7%. Each patient had 1–13 affected lung segments with an average of 4.2 affected lung segments (any segment with multiple lesions was counted only once).

**Fig. 2:**
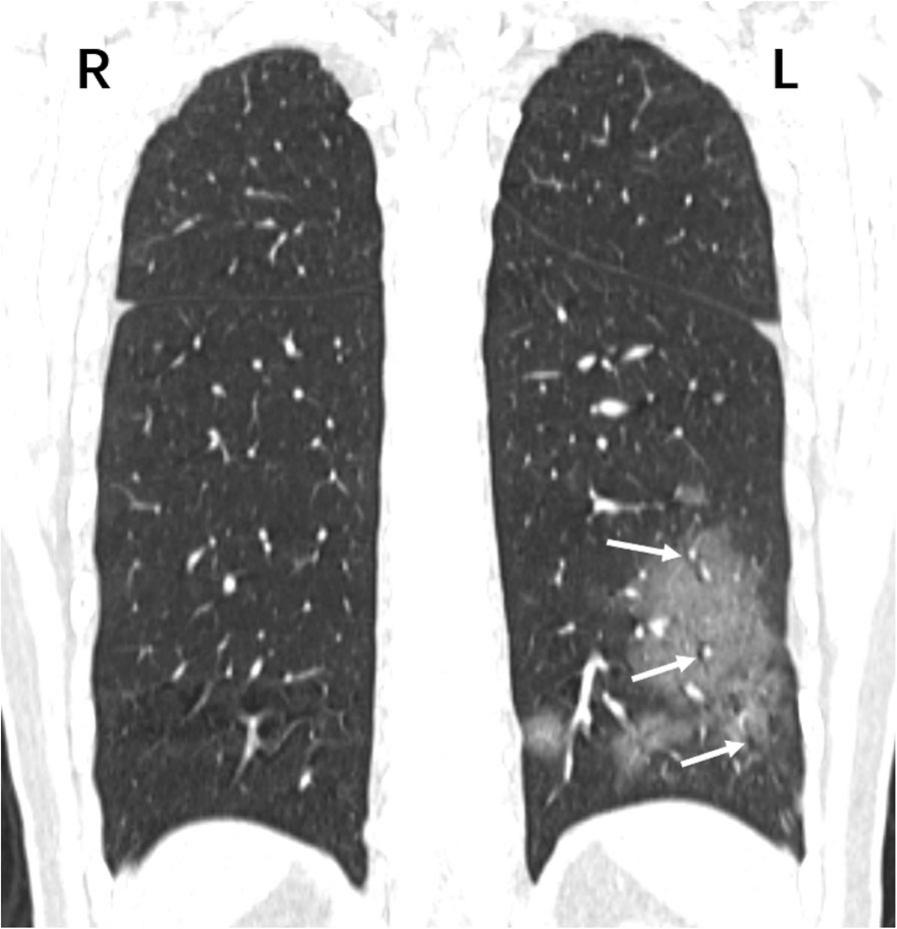
A 39-year-old female patient presenting with fever and cough, and a clear epidemiological history of contact, was admitted to our hospital. CT image shows multiple patchy GGO patterns in the lower lobes of both lungs (here, the reconstructed coronal CT image only shows lesions in the left lung). Long white arrows indicate multiple air-filled bronchi inside the lesions. NAT confirmed that the patient was infected with COVID-19. R = Right, L = Left, GGO = Ground-glass opacity, NAT = Nucleic acid test

**Fig. 3:**
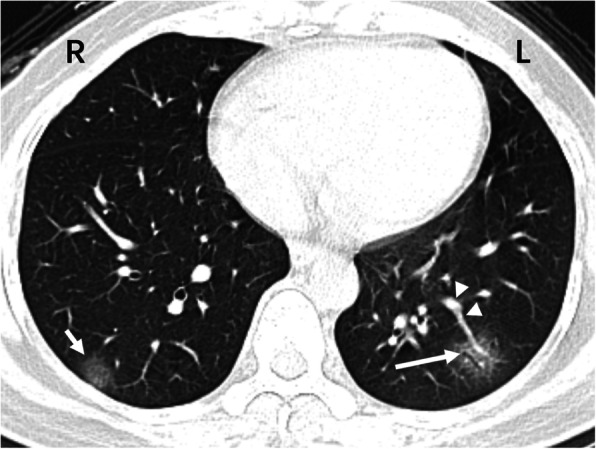
This CT image refers to the same patient (F, 39Y) as Figure 3. Patchy GGO is seen in the posterior basal segment of the left lower lobe and the posterior basal segment of the right lower lobe. The right lung lesions are localized in the peripheral lung field adjacent to the subpleural region (short white arrow). The outermost edge of the left lung lesions is 3.7 mm from the subpleural areas, and there are visible air-filled bronchi (long white arrow) inside the lesion; the white arrowheads show that a vein in the lower left lung has penetrated the lesion. R = Right, L = Left

**Fig. 4:**
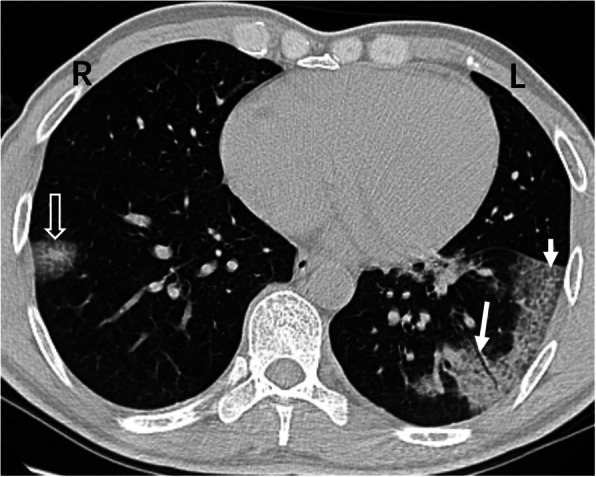
CT image of a 46-year-old male patient showing a combination of patchy GGO with consolidation (long black arrow) in the anterior basal segment of the right lower lobe, and a localized lesion in the peripheral lung field adjacent to the subpleural region. A combination of GGO with consolidation in multiple shapes is seen in the lateral and posterior basal segments of the left lower lobe, where most areas show a crazy-paving appearance (short white arrows), with visible air-filled bronchi (long white arrow) in the lesion. R = Right, L = Left

**Fig. 5:**
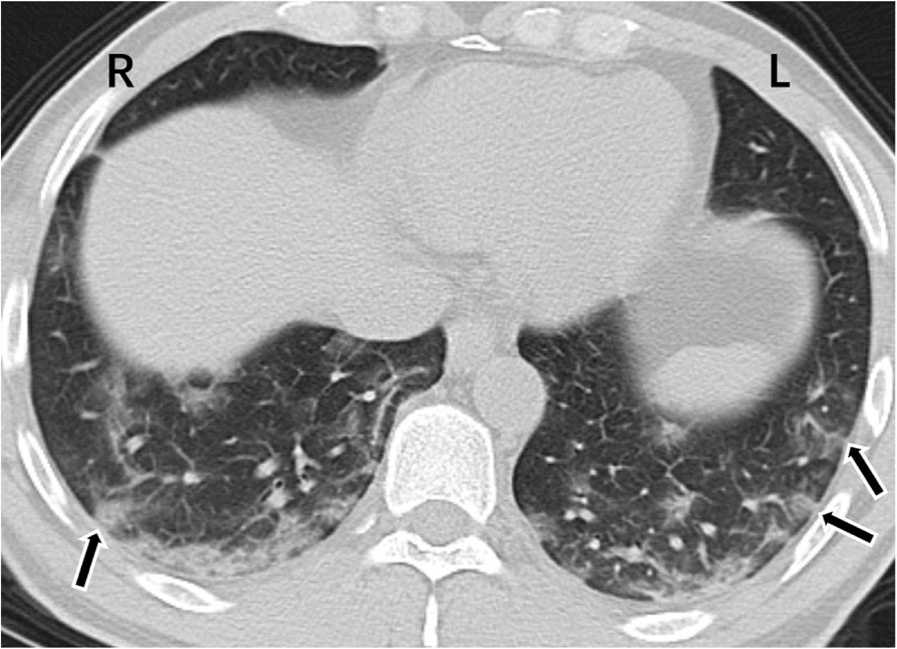
CT image of another 46-year-old male patient showing multiple areas of small patchy GGO on the lateral and posterior basal segments of both lower lobes, with many lesions immediately adjacent to the subpleural region. There is visible localized pleural thickening (long black arrow), but no obvious pleural effusion is observed

Lesions tended to be distributed in the peripheral lung field (73.8%) (Table 2) but were seldom distributed in the central lung field (9.0%). In both groups 1 and 2, patients with involvement of the central zone were few (4 of 51 and 2 of 29 patients in groups 1 and 2, respectively), while many patients showed affected peripheral zone (21 of 51 and 25 of 29 patients in groups 1 and 2, respectively). In the peripheral zone, many lesions were adjacent to the subpleural region (284 of 548 peripheral lung lesions, accounting for 51.8%) (Figs. 2, 3, 4 and 5). In the whole lung field, the near-subpleural lesions accounted for 38.2% (284 of 743 lesions). Furthermore, the number of patients with subpleural involvement was relatively large (28 of 51 and 13 of 29 patients in groups 1 and 2, respectively).

### Size of lesions

As shown in Table 3, small-sized lesions (diameter < 3 cm) were dominant in the lungs (491 of 743 lesions, accounting for 66.1%) of group M patients, which was formed by pooling groups 1 and 2. Compared with group 2 (24 of 181 lesions, 13.3%), group 1 had a higher proportion (219 of 562 lesions, 39.0%) of large-sized lesions (diameter > 3 cm).

**Table 3:** Number of segments with lesions of particular sizes

Lesion Diameter	No. of Segments		
	Group 1	Group 2	Total (Group M)
	(*n* = 562)	(*n* = 181)	(*n* = 743)
< 1 cm	75 (13.3%)	46 (25.4%)	121 (16.3%)
1 < 3 cm	259 (45.6%)	111 (61.3%)	370 (49.8%)
3 cm < 50% of segment	106 (18.9%)	11 (6.1%)	117 (15.7%)
50% of segment or more	113 (20.1%)	13 (7.2%)	126 (17.0%)

Note: Number in parentheses are percentages

### Lesion characteristics in CT images

The lesions mostly presented with GGO (421 of 743 lesions, 56.7%; group M) (Table 4, Figs. 2, 3, and 5) or a combination of GGO with consolidation (288 of 743 lesions, 38.8%; group M) (Fig. 4). Forty-eight of the 51 patients in group 1 and 28 of the 29 patients in group 2 presented with GGO or a combination of GGO with consolidation, while very few patients presented with pure consolidation (25 of 743 lesions, 3.4%; group M). Two of the 51 patients in group 1 and one of the 29 patients in group 2 presented with pure consolidation.

**Table 4:** Number of patients and number of lesions with particular characteristics at thin-section CT

Characteristics	No. of Patients			No. of Lesions
	Group 1	Group 2	Total (Group M)	Total (Group M)
	(*n* = 51)	(*n* = 29)	(*n* = 80)	(*n* = 743)
Opacification				
Ground glass	31	21	52	421 (56.7%)
Consolidation	2	1	3	25 (3.4%)
Mixed ground glass and consolidation	17	7	24	288 (38.8%)
Interstitial thickening				
Interlobular septal	31	13	44	203 (27.3%)
Intralobular	33	18	51	281 (37.8%)
Bronchiectasis	33	11	44	167 (22.5%)
Localized pleural thickening	26	14	40	143 (NA)
Pleural effusion	0	0	0	0 (NA)
Pneumothorax	0	0	0	0 (NA)
Mediastinal lymphadenopathy	1	0	1	1 (NA)

*NA* not applicable

Note: Numbers in parentheses are percentages

It was also observed that many patients had complications with localized pleural thickening (40 of the 80 patients in group M) (Table 4, Fig. 5). Moreover, of the total 743 lesions in group M, some presented with interstitial thickening, including interlobular septal thickening (*n* = 203, 27.3%) and intralobular thickening (*n* = 281, 37.8%) (Table 4). Generally, these types of thickening were superimposed on GGO to generate a crazy-paving appearance (Fig. 5). Air bronchogram was present in 22.5% of the lesions in group M (167 of 743 lesions) (Table 4, Figs. 2, 3 and 4), which was likely to affect the air supply in segmental areas. No patient presented with pleural effusion, pneumothorax, masses, emphysema, lesion cavities, or calcifications. Fibrosis of the lung apex was observed in 11 patients, three of which presented with calcified nodules, indicative of previous tuberculous infection. One patient (a 59-year-old woman) showed unilateral lymph node enlargement in the mediastinum with dumbbell-shaped lymph nodes, and another patient (a 69-year-old man) presented with a 2.1-cm irregular nodule in the right upper lobe. These two patients will be subjected to further evaluation.

## Discussion

Investigations and studies to date [4] have indicated that SARS-CoV-2 is a novel virus of the genus *Betacoronavirus*, with an envelope and circular or oval shape appearance, often polymorphic, with a diameter of 60–140 nm. Its genetic characteristics are markedly different from those of severe acute respiratory syndrome-related coronavirus (SARSr-CoV) [5] and Middle East respiratory syndrome coronavirus (MERS-CoV) [6]. Current research has shown that it has more than 85% homology with a bat SARS-like coronavirus (bat-SL-CoVZC45) [7]. COVID-19 infects human respiratory epithelial cells by binding to human ACE2 through the S-protein [8], and it has been confirmed that it can be transmitted to people via respiratory droplets and close contact [4]. The latest large-sample data (72,314 samples) indicate that the overall case-fatality rate of this disease is about 2.3% [9].

It was observed in this study that COVID-19 had some common manifestations on chest thin-section CT images. Thus, many patients showed the involvement of both lungs (group 1, 82.3%; group 2, 79.1%), and lesions were more distributed in both lower lobes than in other areas (433 of 743 lesions, 58.3%; group M). Lesions tended to be distributed in the peripheral lung areas (548 of 743 lesions, 73.8%; group M), and a considerable number of lesions were located near-subpleural regions, accounting for 38.2% of all lesions. Lesions were usually small (66.1% less than 3 cm in diameter, group M), especially in the early stage of the disease (based on the assumption that patients in group 2 were at an early stage of infection compared to group 1). Most of the lesions presented with GGO and some of them also showed consolidation, while very few lesions presented with pure consolidation. Other typical patterns in the CT images included air bronchogram, interlobular septal thickening, intralobular thickening, and a crazy-paving appearance. Nearly no pleural effusions, pneumothorax, and lymph node enlargement were observed in the CT images.

Chest thin-section CT manifestations of this disease were not diagnosis-specific. Given that SARS-CoV-2 belongs to the same genus, *Betacoronavirus,* as SARS-CoV-1 and MERS-CoV, the results of this study were compared with those of other studies (Table 5). Some CT manifestations of SARS in the study by Wong et al. [10], were similar to those of COVID-19 in this study, such as the preferential distribution of lesions in the periphery and lower lobes of the lungs, presence of multiple areas with GGO, and the frequent presence of interlobular septal and intralobular thickening. However, the study by Wong et al. [10], reported a lower number of lesions per patient on average than the current study on COVID-19 patients. Moreover, the previous study did not mention or imply the distribution of lesions in the peripheral near-subpleural region; it also did not mention complications with localized pleural thickening. However, in the present study, a considerable number of patients in the case-combined group (i.e., group M) presented with lesions in the peripheral near-subpleural region, and additionally, a considerable number of these patients also presented with localized pleural thickening. Moreover, MERS, which is also a coronavirus infection, is slightly similar to COVID-19 in terms of the CT features. For example, it was reported that the main CT features of MERS were GGO (53%) or consolidation (20%), with 33% of the patients showing a combination of GGO with consolidation and some even displaying a crazy-paving appearance [11]. In an X-ray-based study of MERS [12], MERS lesions were mainly distributed in the peripheral middle and lower lobes, with unifocal involvement (69%) being more common than multifocal involvement (31%); the number of patients with pleural effusion was as high as 63.2% in the deceased group, but only 13.9% in the recovered group. Moreover, another related CT study [13] showed a similarly high number (33%) of patients with pleural effusions. MERS is often complicated with a significantly higher incidence (16.4%) of pneumothorax compared with COVID-19. These CT features are significantly different from those of COVID-19 observed in this study. Das et al. [12], proposed that pleural effusion combined with other risk factors can be considered as a significant predictor of prognosis for patients, which is only partially supported by this study, as none of the 80 patients admitted to our hospital has died to date (34 have been cured and discharged), and none had developed pleural effusion and pneumothorax. Such discrepancy between the study of Das et al. [12], and the present study may be partially attributed to higher mortality (44%) in MERS-CoV [11]. However, it is evident that the manifestations of COVID-19 are significantly different from those of MERS, at least in terms of CT features.

**Table 5:** The difference of chest CT findings between covid19 pneumonia and other coronavirus infection

Typical CT Findings									
Name of viral pneumonia	Typical CT Findings								
	Distribution	Lesions characteristics	Crazy-paving appearance	GGO	Consolidation	Bronchiectasis	Localized pleural thickening	Pleural effusion	Pneumothorax
COVID-19	Lower lobes and peripheral zone, A considerable number of lesions were located near-subpleural regions	GGO,Consolidation, Mixed GGO and consolidation	Variable	+++	Rare	+	common	Rare	Rare
SARSr-CoV	Lower lobes and peripheral zone	GGO,Consolidation, Mixed GGO and consolidation	Variable	+++	+	Rare	Not definite	Rare	Rare
MERS-CoV	Middle and lower lobes, Peripheral zone	GGO,Consolidation, Mixed GGO and consolidation	Variable	+++	+	+	Not definite	+	++

Note. *COVID-19* 2019 Corona Virus Disease, *SARSr-CoV* Severe Acute Respiratory Syndrome related Coronavirus, *MERS-CoV* Middle East respiratory syndrome coronavirus

Probability of being observed: Rare = Less than 10%, + = 10–25%, ++ = 25–50%, +++ =50%–75, ++++ = Greater than 75%

A crazy-paving appearance in thin-section CT images refers to a line-like pattern superimposed on the GGO background, which resembles irregular paving stones but is not a specific radiological sign. The prevalence of crazy-paving appearance is 100% in patients with alveolar proteinosis, 67% with diffuse alveolar injury, 31% with acute interstitial pneumonia, and 21% with adult respiratory distress syndrome [14]. A crazy-paving appearance may also be observed in patients with radiation pneumonia or organizing pneumonia (OP). Although radiological assessment alone does not establish the cause of the disease, it can be used in combination with other radiological findings. Furthermore, the relationship between CT features and clinical symptoms could be exploited to determine in most cases whether a lung lesion is caused by COVID-19 infection based on the differences of the spatial location among different types of lung lesions, especially in patients who have an epidemiological history of contact. OP, formerly known as bronchiolitis obliterans with organizing pneumonia (BOOP), has CT features partially similar to those of COVID-19. The similar CT features include lesion distribution in the lower lobes and the peripheral region, GGO, consolidation, crazy-paving appearances, and air bronchogram [15]. In this study, only a small proportion of COVID-19 lesions presented with consolidation (25 of 743 lesions, 3.4%, group M), but consolidation is relatively common in OP patients (31.6%) [16] and the proportion may even be as high as 70% [15]. Patients with OP also present with lymph node enlargement (13%) and pleural effusion (20%) [17]. However, no pleural effusion was observed in the COVID-19 patients of this study, and only one patient presented with lymph node enlargement, while its relevance to COVID-19 remains to be further assessed. In addition, patients with chronic eosinophilic pneumonia and acute extrinsic allergic alveolitis also commonly present with GGO and consolidation, which are likely accompanied by a crazy-paving appearance. However, a high prevalence (74.4%) of consolidation in patients with chronic eosinophilic pneumonia was observed in a chest thin-section study [16], and opacity changes mainly exist in the upper lobes and the peripheral lung field [18]. Extrinsic allergic alveolitis is caused by an abnormal immune response to inhaled allergens, but its lesions are mainly distributed in the central region of the lungs and the center of the lobules, with the possible presence of nodules [19, 20].

During the outbreak of COVID-19, the 30 patients in group 3 showed mild symptoms with normal chest radiographs. If the CCDC guidelines were to be rigorously followed, CT scans should not have been performed on this group of patients. Some of them were suspected of having a history of close contact, while a few were medical staff infected during the implementation of treatment measures, with high anxiety observed in some patients. Although CT is considered unnecessary for these patients, the CT findings are indeed useful for assisting with understanding of the disease.

The present study is subject to some limitations. Firstly, due to the limited number of patients enrolled in the study, the existing CT results may fail to reveal the complete distribution and appearances of COVID-19 lesions. Secondly, this study failed to comparatively investigate the changes in CT features during the course of the disease, which would have provided important insights into this disease. These limitations are attributed to the fact that most patients are still undergoing treatment, while only 34 patients have been cured and discharged, making it currently impossible to fully collect the results of the CT rescans. CT rescans will be included in future CT studies on COVID-19.

## Conclusion

The common features of chest thin-section CT of COVID-19 were multiple areas of GGO, which were sometimes accompanied by consolidation. The lesions were mainly distributed in the lower lobes and peripheral zone, and a large proportion of peripheral lesions were accompanied by localized pleural thickening adjacent to the subpleural region. There was no pleural effusion or pneumothorax, and there was almost no lymph node enlargement.

## Data Availability

The datasets used and/or analysed during the current study available from the corresponding author on reasonable request.

## Abbreviations

COVID-19: Coronavirus disease-2019
CT: Computed Tomography
CCDC: Chinese Center for Disease Control and Prevention
SARSr-CoV: Severe acute respiratory syndrome-related coronavirus
MERS-CoV: Middle East respiratory syndrome coronavirus
ACE2: Angiotensin-converting enzyme 2
WHO: World Health Organization
NAT: Nucleic acid testing
RT-PCR: Reverse transcription-polymerase chain reaction
GGO: Round glass opacification
OP: Organizing pneumonia
BOOP: Bronchiolitis obliterans with organizing pneumonia

## References

[1] WHO. Coronavirus disease (COVID-19): situation report-55: World Health Organization; 2020. https://www.who.int/docs/default-source/coronaviruse/situation-reports/20200315-sitrep-55-covid-19.pdf?sfvrsn=33daa5cb_6.

[2] Imai N, Dorigatti I, Cori A, Riley S, Ferguson NM. Estimating the potential total number of novel coronavirus cases in Wuhan City, China, China Jan 25, 2020. https://www.imperial.ac.uk/mrc-global-infectious-disease-analysis/news--wuhan-coronavirus/.

[3] National Health Commission of the PRC. Diagnostic quality scheme for novel coronavirus pneumonia, 5th (Revised Version). 2020 Feb. Available from: http://www.gov.cn/zhengce/zhengceku/2020-02/09/5476407/files/765d1e65b7d1443081053c29ad37fb07.pdf.

[4] Huang C, Wang Y, Li X (2020). Clinical features of patients infected with 2019 novel coronavirus in Wuhan, China. Lancet.

[5] Wan Y, Shang J, Graham R (2020). Receptor recognition by novel coronavirus from Wuhan: an analysis based on decade-long structural studies of SARS. J Virol.

[6] de Groot RJ, Baker SC, Baric RS (2013). Middle East respiratory syndrome coronavirus (MERS-CoV): announcement of the coronavirus study group. J Virol.

[7] Zhu N, Zhang D, Wang W (2020). A novel coronavirus from patients with pneumonia in China, 2019. N Engl J of Med.

[8] Chen Y, Liu Q, Guo D (2020). Emerging coronaviruses: genome structure, replication, and pathogenesis. J Med Virol.

[9] The Novel Coronavirus Pneumonia Emergency Response Epidemiology Team (2020). The epidemiological characteristics of an outbreak of 2019 novel coronavirus diseases (COVID-19) in China. Zhonghua Liu Xing Bing Xue Za Zhi.

[10] Wong KT, Antonio GE, Hui DSC (2003). Thin-section CT of severe acute respiratory syndrome: evaluation of 73 patients exposed to or with the disease. Radiology.

[11] Das KM, Lee EY, Langer RD (2016). Middle East respiratory syndrome coronavirus: what does a radiologist need to know?. AJR Am J Roentgenol.

[12] Das KM, Lee EY, Al Jawder SE (2015). Acute Middle East respiratory syndrome coronavirus: temporal lung changes observed on the chest radiographs of 55 patients. AJR Am J Roentgenol.

[13] Das KM, Lee EY, Enani MA (2014). CT correlation with outcomes in 15 patients with acute Middle East respiratory syndrome coronavirus. *AJR Am J Roentgenol* 2015;204(4):736–42. Doi: 10.2214/AJR.14.13671.Baque-Juston M, Pellegrin a, Leroy S, et al. organizing pneumonia: what is it? A conceptual approach and pictorial review. Diagn Interv Imaging.

[14] Arakawa H, Kurihara Y, Niimi H (2001). Bronchiolitis obliterans with organizing pneumonia versus chronic eosinophilic pneumonia. AJR Am J Roentgenol.

[15] Preidler KW, Szolar DM, Moelleken S (1996). Distribution pattern of computed tomography findings in patients with bronchiolitis obliterans organizing pneumonia. Investig Radiol.

[16] Stoller JK, Broaddus VC (2016). Eosinophilic lung diseases. Murray and Nadel’s Textbook of Respiratory Medicine.

[17] Silver SF, Muller NL, Miller RR (1989). Hypersensitivity pneumonitis: evaluation with CT. Radiology.

[18] Amansakhedov RB, Limarova IV, Perfiliev AV, et al. Comparative analysis of the semiotics of disseminated pulmonary tuberculosis and exogenous allergic alveolitis in accordance with the data of computed tomography. Vestn Rentgenol Radiol. 2016;97(2):79–84. 10.20862/0042-4676-2016-97-2-79-84. 10.20862/0042-4676-2016-97-2-79-84 27522702

